# Paternal early experiences influence infant development through non-social mechanisms in Rhesus Macaques

**DOI:** 10.1186/1742-9994-12-S1-S14

**Published:** 2015-08-24

**Authors:** Erin L Kinnally, John P Capitanio

**Affiliations:** 1 California National Primate Research Center, University of California Davis, CA, 95616; 2 Department of Psychology, University of California, Davis, CA 95616

**Keywords:** paternal line, early life stress, rhesus macaque, nursery rearing

## Abstract

**Background:**

Early experiences influence the developing organism, with lifelong and potentially adaptive consequences. It has recently become clear that the effects of early experiences are not limited to the exposed generation, but can influence physiological and behavioral traits in the next generation. Mechanisms of transgenerational effects of parental early experiences on offspring development are often attributed to prenatal or postnatal parental influence, but recent data suggest that germ-line plasticity may also play a role in the transgenerational effects of early experiences. These non-genetic transgenerational effects are a potentially important developmental and evolutionary force, but the effects of parental experiences on behavior and physiology are not well understood in socially complex primates. In the non-human primate, the rhesus macaque, nursery rearing (NR) is an early life manipulation used for colony management purposes, and involves separating infants from parents early in life. We examined the effects of maternal and paternal early NR on infant rhesus macaque immunity, physiology, and behavior.

**Results:**

We theorized that differences in behavior or physiology in the absence of parent-offspring social contact would point to biological and perhaps germ-line, rather than social, mechanisms of effect. Thus, all subjects were themselves NR. Male and female infant rhesus macaques (N= 206) were separated from parents and social groups in the first four days of life to undergo NR. These infants differed only in their degree of NR ancestry – whether their dams or sires were themselves NR. At 3-4 months of age, infants underwent a standardized biobehavioral assessment. Factors describing immunity, plasma cortisol, and emotion regulation were generated from these data using factor analysis. Paternal, but not maternal, NR was associated with greater emotionality and higher plasma cortisol, compared with infants born to CONTROL reared fathers.

**Conclusions:**

These data suggest that macaque biobehavioral makeup is strongly influenced by paternal experiences, and via non-social mechanisms.

## Introduction

Early experiences re-organize somatic and neurobehavioral development, and can lead to shifts in physiological stress response [1,2], immunity and health [3-6] and behavioral traits[1,3,7]. It has recently become clear that the reorganization of these systems may not be limited to the exposed generation, but may be observed in subsequent generations through the maternal and/or paternal line[8-14]. These non-genetic transgenerational effects of early experiences are a potentially important developmental and evolutionary force, but the effects of parental experiences on behavior and physiology are not well understood in socially complex primates.

In mammals, the disruption of normative early attachment relationships, which can occur in cases of poor parental care, social separation, or neglect, can be particularly formative for the developing infant[3,4,8]. These experiences can shift physiological and behavioral stress response [2], sociality [7], and disease risk [3-6], all of which are central components to an individual's fitness. It has recently become clear that poor quality attachment relationships early in life can also influence neurobehavioral development in subsequent generations [8-14]. These transgenerational effects of adversity have often been attributed to environmental transmission, as can occur through generational cycles of parent-infant attachment or social learning[10-15]. Biological mechanisms for such phenomena, in contrast, have been thought to be few.

This is the case largely because, until the last decade, the only non-social mode of inheritance from parents was believed to be genetic, which should be unchanged by experiences. However, non-genetic germ-line mechanisms for the transgenerational effects of early experiences have been recently identified [16,17]. Epigenetic changes to sperm DNA methylation patterns or micro-RNA expression, rather than changes to the genome sequence itself, have been observed in the sperm of fathers that experience temporary stress, coinciding with enhanced anxiety-related behavior in fathers [18-24]. Intriguingly, these changes can be inherited by the next generation: the epigenetic differences observed in sperm between control and stressed fathers are also observed the in brains of their offspring[18-20]. Intriguingly, this inheritance predicts phenotype: these epigenetic profiles inherited from stressed fathers predict anxiety-related traits in offspring that themselves were not exposed to stress. Of course, this is not always the case. Several studies have demonstrated a role for maternal factors, including maternal behavior, in the effects of paternal stress on infant outcomes [19,25]. If transmission of experience-related traits between generations is observed between individuals without regular social contact between offspring and parents however, this evidence may point to germ-line mechanisms of inheritance of experience-related traits.

While there is some evidence in humans that early experiences may affect subsequent generations [26,27], it is not yet known whether germ-line factors play a role in transgenerational effects of early experiences in primates, including humans. In the present study, we investigated whether the effects of early life experiences in one generation would be observed in a subsequent generation of rhesus macaques, in the context of limited social contact between the two generations. Rhesus macaques are a useful species to help us understand the generality of the phenomenon across primates, because they show genetic and neural homology and comparable social complexity with other old-world primates, including humans [28,29].One of the best-characterized experimental early life manipulations in macaques is peer-rearing or nursery-rearing, [NR; 30, 31].NR monkeys can be compared with subjects that are raised by their mothers in extended social groups [CONTROL rearing), as is typical in macaque life history. Typically, NR infants are removed from their mothers on the first days of life, and then, usually within about a month, are housed with peers. One of the important consequences of NR is reorganization of the immune, endocrine, and nervous systems. Compared with CONTROL animals, NR macaques are characterized by widespread reorganization of stress pathway genes [31-33], production of fewer immune cells [34], and greater emotionality, such as the expression of emotion-related behaviors in response to challenge, [such as fear grimaces, vocalizations, and lip smacks in macaques;[35], and altered regulation of the hypothalamic-pituitary-adrenal (HPA) axis [2,30,36], outcomes that are similar to those seen in humans that experience early adversity[1,3,5-7,9].While NR can alter developmental trajectories, these outcomes do not necessarily impair the ability of NR animals to have relatively normal social lives or to reproduce later in life. Thus, when NR animals breed, we can trace the effects of NR on offspring development and compare them with offspring of CONTROL reared macaques.

In the present report, we investigated whether maternal and/or paternal early experiences influenced infant immunological, physiological, and behavioral development in a non-human primate, the rhesus macaque. To do so, we compared the biobehavioral development of NR infants (3-4 months of age) based upon the their paternal and maternal rearing history – their dams and sires had been either CONTROL-reared or NR. We measured multiple behavioral, physiological, and immunological responses to challenge in these offspring. To test the hypothesis that transgenerational effects of NR were mediated through non-social mechanisms, our subjects were all themselves NR, which limited the opportunity for social transmission of any effects of parental NR to their offspring.

## Results

**Figure 1:**
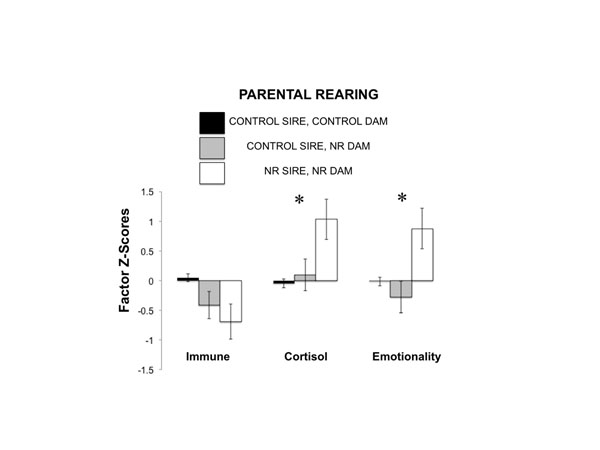
Paternal NR is associated with higher plasma cortisol factor scores and greater trait Emotionality factor scores. Weighted means are presented ± standard error of the mean. *p < .05

We investigated whether maternal and paternal rearing would influence immune, physiological, and behavioral outcomes in their offspring. In the analysis, we controlled for potential contributing factors, including pedigree, infant sex, cohort (year of testing), paternal and maternal years of age, and day of maternal NR in four cases when mothers were not NR on Day 1. Paternal rearing history significantly predicted male and female infant measures (F_3, 194_ = 3.040, p = 0.030, partial eta^2^= 0.045; See Figure 1). The effects were such that infants with NR fathers exhibited significantly greater emotionality factor scores, which encompassed emotionality-related behaviors on days 1 and 2 of the assessment (F_1, 196_= 7.393, p = 0.007, partial eta^2^= 0.036) and higher plasma cortisol factor scores, which encompassed plasma cortisol sampled on four occasions: 2 hours post social separation, 9 hours post separation, post dexamethasone treatment and post-ACTH treatment, (F_1, 203_ = 4.958, p = 0.027, partial eta^2^= 0.025) compared to infants with CONTROL reared fathers. Paternal NR did not significantly predict immune factor scores, which comprised white blood cell count, lymphocyte count, CD4+ cell count, and CD+ cell count, (F_1, 203_ = 0.586, p = 0.445, partial eta^2^= 0.025). Maternal rearing history did not significantly predict physiological or behavioral measures (F_3, 194_ = 1.475 p = 0.223, partial eta^2^= 0.003), nor did sex, pedigree, paternal or maternal age, or testing cohort(all p > .05).Notably, day of maternal separation from mother did not predict infant outcomes (p > .05). Post-hoc analysis demonstrates that there was no interaction between paternal rearing and infant sex (p> .05).

## Discussion

Early life experiences reorganize the physiology [1,2] immunity[3-6], and behavior [1,4-6]of the exposed individual, with lifelong consequences. Our data extend previous studies that have demonstrated that the experience of NR in macaques is associated with reduced immune cell counts[34], dysregulated plasma cortisol [2,30,36]and greater emotion-related behavior[35]. We now demonstrate that some of these effects are also observed in offspring of NR males. Male and female offspring of NR males exhibited greater trait emotionality and higher plasma cortisol compared with descendants of CONTROL males. Notably, these differences were observed in the absence of extended social exposure to fathers, mothers, or extended families and social groups, as our infant subjects were all themselves NR. It is unlikely that genetic influences explain these results, because parental NR was administered randomly, our population pairwise relatedness was low(less than 2%), and we controlled for pedigree in our analysis. In sum, our work suggests that vestiges of early stress may not be limited to exposed individuals in primates, but may influence subsequent generations through the paternal line via apparently non-social mechanisms.

In the present study, paternal NR predicted greater emotionality and higher plasma cortisol in NR offspring. Maternal effects of NR were not observed in this study. These findings are consistent with several studies that have demonstrated paternal line effects of adversity on anxiety-and health-related traits in offspring, even in the absence of social contact with fathers [18-23], but are somewhat inconsistent with rodent studies that have demonstrated equivalent transgenerational effects of both paternal and maternal exposures[23]. This does not necessarily mean that maternal NR has no effect on offspring development, but its effects may be more complex to detect. Traits inherited via the maternal line arise from interactions among maternal genetics, prenatal factors, and postnatal investment. In the present study, the impact of postnatal investment would have been minimal: mothers and infant subjects remained together usually less than one full day (but no more than four days) after infant birth. But it is possible that maternal NR interacted with maternal genetics and prenatal factors in a way that obscured the effects of maternal rearing history on infant development in this analysis. Another possible source of heterogeneity is that, while all fathers were NR on Day 1 of life, as were the majority of NR mothers, a small subset (4/27) mothers had been separated from their own mothers later in development (two by Day 3 of life, but one on Day 23 and the other on day 85). We controlled for maternal postnatal day NR in our analysis. This variable did not affect out results, suggesting that the number of days that female infants remained with mothers before being NR did not change the effects on offspring. However, due to the small sample size of our NR mothers, it is possible that this heterogeneity in NR practices affected our results. To address this potential issue, we re-analyzed the data without these four females, and the results were identical. Thus, we believe that the main effects of maternal NR are, in fact, weaker than that of paternal NR, but future studies must corroborate this finding.

Because the effects we observed were specific to the paternal line, we considered the possibility that parental NR may be sex-specific, and thus, affect males and females differently. While other studies have demonstrated sex differences in sensitivity to experiences[37,38], and more recently it has been discovered that there may be sex differences in the effects of parental environmental exposure as well [18,23-27], this was not the case in our population. Males and females were similarly affected by paternal line NR in our study, but it remains to be determined whether affected males and females will equally transmit the effects of paternal NR to their own offspring.

The mechanisms for the effects of paternal early stress on offspring trait emotionality and plasma cortisol are not yet known. The potential for social or environmental mechanisms to explain the effects of paternal NR in this study is limited. Thus, the effects of paternal NR may arise from germ-line mechanisms. If germ line elements were changed in males following early experiences and were stably inherited by progeny, this would help explain the effects of paternal NR. Presumably substantial changes to the DNA sequence itself are not widespread in sperm following stress, but epigenetic signals, such as histone modifications, micro RNAs, hormones/transcription factors, and DNA methylation patterns are all present in paternal germ cells and may be inherited by the developing infant [18-20].While several recent studies in rodents have demonstrated such transgenerational inheritance of exposure-induced DNA methylation patterns and micro RNA expression via sperm[18-23], it is not yet known if such acquired germ-line changes are inherited in primates, including humans.

Why would paternal, but not maternal, NR effects be inherited through germ line mechanisms? Because most mammalian fathers, including rhesus macaques, contribute little more than germ cells to progeny (monogamous, biparental species represent an exception among mammals[39,40]), it has been theorized that we might expect such germ-line mediated mechanisms to evolve, to allow for intergenerational transmission of acquired information between males and their descendants in the species-typical absence of social contact [41-43]. We would not necessarily expect such germ line mechanisms for transmission to arise between mother and offspring in mammals, because there are other opportunities for such mother-infant transmission, including prenatal and postnatal signaling.

An alternate explanation for our findings is that mothers may adjust their prenatal investment in infants based on NR-related qualities of the father. Avian and mammalian mothers have been shown to adjust postnatal investment in infants based on paternal characteristics. In one recent study, female mice that were mated with males from impoverished early life conditions negatively adjusted their postnatal maternal investment toward offspring, which corresponded in a linear fashion with the rate of anxiety-related behaviors exhibited by the father. This reduction in maternal care, in turn, predicted offspring growth[28]. This study was consistent with the ethological literature that suggests that females may invest differently in offspring based on perceived characteristics of the male mate, for example, in mallards[44]. In these previously described conditions, however, the female mated with only one male and therefore the identity of the father was likely known to the female. Macaques are a polygynous species, and females mate with multiple males throughout the breeding season. While evidence is scarce, there are few data available to suggest that females are aware of the identities of their offspring's fathers in natural group-living conditions. Similarly, in the present study, over 60% of our subjects’ mothers lived in conditions with multiple males, meaning that mothers most likely did not know the identity of offspring's fathers. Future studies must investigate this possibility more systematically, however.

These findings have important evolutionary implications. When adverse early experience in a previous generation is associated with an emotionally reactive temperament and heightened cortisol in subsequent generations, this parental programming may influence the fitness and reproductive success of future generations. Initially, it may seem that the transgenerational programming of such traits may be disadvantageous to descendants’ survival and reproductive success, especially if it leads to poorer mental and physical health. One might expect that a compensatory effect of parental environmental exposures on the health of their descendants would be a more adaptive strategy in an evolutionary context. Such a compensatory effect has been observed in humans [26,27]. We provide some evidence for a compensatory effect as well. A previous study in this population demonstrated that NR predicts dampened cortisol output in 3-4 month olds. Our NR infants with NR fathers still exhibit dampened cortisol compared with CONTROL reared infants, but their cortisol was merely higher than other NR infants of CONTROL fathers. These results may suggest that the effects of NR on cortisol are somewhat, although not completely, reversed, in the next generation. It is also possible that challenges in a previous generation shape adaptive coping strategies in the next generation. For this to be an advantageous strategy, one might expect that transgenerational programming should optimize fitness under parental environmental conditions. To our knowledge, this has not yet been investigated, but at least two studies have demonstrated that early adversity may actually optimize survival skills and reproductive strategies in the same generation. Rats that received poor maternal care as infants reach puberty earlier and engage in higher rates of sexual behavior as adults, perhaps ensuring reproductive success in uncertain conditions [45]. Additionally, while rats that received lower maternal care in infancy perform worse on a learning task than infants that received better care, these low care infants perform better than high care rats under stressful conditions, suggesting that early stress may have optimized performance for stress[46].Thus, transgenerational inheritance of stress coping strategies may actually optimize stress adaptation in the next generation – particularly if the offspring's environment matches the stressed parent's environment. Future studies will examine whether transgenerational stress programming may confer an advantage in subsequent generations in primates.

There were several important limitations of this study. The first limitation is that conditions similar to NR are not likely to occur in the wild, as infants that survive after being separated from parents have likely received care from other adult group members rather than peers. Thus, the ecological relevance of this paradigm is somewhat limited. However, we suggest that shifts in immunity, physiology, and behavior following variation in early experiences are likely facilitated by genomic, neural, and physiological shifts. Thus, the specific characteristics of the early experience may not be as important to the ecological relevance of our results as is the demonstration that these systems show shifts in response to different types of experiences. NR has been linked with similar biobehavioral shifts compared with other types of early experiences in humans and other animals [1-3,5-7,9], supporting its generalizability. Certainly, other types of early experiences with similar effects on behavior and physiology may have been transmitted across generations, as others and we have previously demonstrated [9-14]. Therefore, while we expect that other types of more ecologically relevant experiences would be subject to transgenerational inheritance, just as we have demonstrated with NR, more research is required. Similarly, another limitation of this study is that we restricted our analysis to offspring that were themselves NR. This approach was taken to eliminate the impact of social mechanisms involved with transgenerational stress effects, but we still do not know whether the effects would be as strong in infants raised in species-typical environments with their parents and social groups. Future studies must bear this out.

A methodological limitation of this study is that, because we assessed physiology and behavior at one time point [3-4 months of age), the lifetime effects of NR ancestry on behavior, physiology, and health, and the relevance to fitness outcomes, are not yet known. We believe that these measures are indicators of future outcomes, however, as previous work has linked these biobehavioral measures with critical health and social outcomes later in life. For example, macaques with biobehavioral profiles suggesting greater anxiety display airway hyper-responsiveness, a predictor of asthma, in adolescence[47,48]. There are also social consequences associated with these measures: in adolescence, macaques tend to prefer to spend time with individuals with similar behavioral traits [49,50], indicating that these traits measured early in life may influence social relationships and even mating preferences. These studies hint that the transgenerational inheritance of anxiety-related traits may impact important aspects of macaque social life and health, but future studies will investigate the continuity of effects into adulthood and ultimate effects on longevity and reproductive success.

In summary, we demonstrate that some of the effects of NR are transmitted to the second generation through non-social mechanisms via the paternal line in rhesus macaques. Stress related traits and diseases, such as cardiovascular disease, metabolic disease, inflammation, and psychiatric illness, have long been known to run in families. This phenomenon has historically been attributed to genetic causes. Our finding that an early experience influences subsequent generations through the paternal line in primates contributes to a growing literature that suggests that some *acquired *experience-related traits may also be “inherited” in mammals, including primates. Future studies will focus on understanding the likely complex social and germ line mechanisms of the effects of paternal-line NR in primates, and their consequences for health, reproductive success, and natural selection.

## Methods

## Subjects

Male (n= 73) and female (n=133) infant (90-120 days of age) rhesus macaques were included in this study. All subjects were specific pathogen free (SPF), meaning that animals were free of Herpes B virus, and were reared indoors (see below for details on rearing conditions) at the California National Primate Center (CNPRC) between the years 2001-2010. Average pairwise relatedness amongst our 206 subjects was low, less than 2%.

## Rearing Conditions

Our subjects were born to mothers living in one of three conditions at the CNPRC: large outdoor social enclosures (52.4%), indoor housing (39.8%), or small outdoor social enclosures (7.8%). For colony management purposes, NR individuals were separated from their mothers between days one and four of life(day 1: 88.3%, Day 2: 5.8%; Day 3: 4.9%; Day 4: 1%) and were relocated indoors. NR infants were housed in an incubator for approximately the first month of life to ensure infants regulate their own temperature. They had *ad libitum* access to formula and substantial human contact. Following this month, and until biobehavioral assessment, infants were housed in indoor individual cages (.46 x .61 x .69 m) with continuous access to a sex- and age-matched pair-mate. At this time, animals were weaned onto monkey chow (Purina, St. Louis, MO).During NR, animals receive about 1–1.25 hr per day of human contact during the incubator phase, and approximately half of that once they were paired with other infants. Human contact occurs for feedings, cage change and sanitation, and daily weighing.

## Parental Rearing

Parents of our subjects had been reared in either CONTROL or NR conditions[2,32]. CONTROL conditions at the CNPRC have been described previously [2,32].These conditions include housing in one of six half-acre outdoor enclosures containing social groups [34-160 macaques) with individuals from at least 6 genetically distinct matrilines with extended kin networks. NR conditions were similar to that experienced by our subjects, except that in four cases, mothers had been separated from their own mothers after the day of birth[2, 3, 23, and 85 days). All fathers underwent NR on Day 1 of life.

Of our 206 NR infant subjects, 179 had both CONTROL reared mothers and fathers, 16 had an NR mother and CONTROL reared father, and 11 had both an NR mother and an NR father. There were no subjects available that had a CONTROL reared mother and NR father.

## Biobehavioral Assessment

All subjects were assessed for biobehavioral traits during a standardized biobehavioral assessment (BBA) at the CNPRC between 2001 and 2010. During a 25-hour relocation and separation from mothers and pair mates, multiple behavioral (activity, emotionality, novel object interaction, temperament ratings), physiological (plasma cortisol at four time points), and immune (white blood cell count, CD 4 + and CD 8 + cell counts) measures are collected from infant subjects. Standardized procedures were designed to ensure that each subject had comparable experiences to all other subjects who underwent assessment. These procedures have been described in detail elsewhere [2,32,51], and not all measures collected were considered in the present study.

Briefly, subjects were captured by CNPRC staff from their pair housing condition and relocated to indoor individual housing (.81 m x .61 m x .66 m) in a temperature-controlled room under a 12:12 hr light/dark cycle by 9AM. For plasma cortisol measures, blood was sampled via femoral venipuncture four times over the 25-hour period, and each sample was decanted into EDTA-treated collection vials. The first sample was collected at 1100 h (AM sample), approximately 2 hrs following social separation/relocation. The second blood sample was collected approximately 5.0 hours after the first sample at 1600 h (PM sample). Subjects were then immediately injected I.M. with dexamethasone (500 ug/kg, American Regent Laboratories, Shirley, NY). The next blood sample was taken via femoral venipuncture 16.5 hours later, at 0830 h (DEX sample). Following this sample, animals were injected I.M. with 2.5 IU Adrenocorticotropic hormone and blood was sampled 30 minutes later (ACTH sample). Cortisol is measured in each of these samples using radioimmunoassay as described previously [3]. Immune cells counts (white blood cells, lymphocytes, CD4+ and CD8+ cells) were counted from AM samples as previously described [52].

Within two hours of relocation, and again the following morning before return to their pair-mates, infants were observed for five minutes and frequency and duration of a variety of emotion and activity-related behaviors were recorded. Exploratory and confirmatory factor analysis [details in 51] was applied to detect latent variables underlying these behaviors. As described previously, factors describing emotionality on Days 1 and 2 were identified [51].

## Data Analysis

All analyses were conducted using SPSS version 22. Three separate factor analyses were conducted to describe the common factors underlying 1.) immune cell counts (white blood cells, lymphocytes, CD 4 + and CD 8 + cells), 2.) parameters of the HPA system (plasma cortisol from AM, PM, DEX and ACTH sampling), and 3) emotionality factor scores on day 1 and 2 of biobehavioral assessment. Factor loadings of 0.4 or greater were considered to contribute significantly to the model, and factor scores were generated using the regression method. A single immune cell factor (variance explained = 82.133%) positively loaded lymphocyte counts, white blood cell counts, CD4+ and CD8+ cell counts. For the cortisol factor (variance explained = 78.198%), all sample measures loaded on this factor positively, meaning that animals with higher AM, PM, DEX, and ACTH had higher cortisol factor scores. The emotionality factor (variance explained = 70.299%) positively loaded emotionality scores on Days 1 and 2.

We used multivariate analysis to determine the relative contributions of paternal and maternal rearing history on infant immune cell counts, plasma cortisol, and emotionality factor scores. Infant sex, cohort (testing year), paternal and maternal age at infant birth, and maternal NR day of separation were included as covariates in the analysis. We controlled for genetic relatedness by entering the 5-digit identification numbers of mothers and fathers as covariates. Identification numbers are assigned in increasing order with each new animal born, so older animals have lower numbers and younger animals have higher numbers. Main effects of paternal and maternal NR, as well as of covariates, were tested. The interaction between maternal and paternal NR could not be tested because there were no subjects that had a CONTROL reared mother and NR father. We also examined these relationships using linear mixed models with sire and dam ID entered as random effects. The results did not differ, so in order to include a simultaneous analysis of all dependent variables, we report only the multivariate analyses. Significance level was set at p ≤ 0.05.

## Ethical Note

All research was conducted in accordance with U.S. federal guidelines and was approved by Institutional Animal Care and Use Committee at UC Davis.

## Declarations

Publication costs for this article were funded by the German Research Foundation (FOR 1232) and the Open Access Publication Fund of Bielefeld and Muenster University.

## Competing interests

The authors have no competing interests to declare.

## Author's Contributions

E.L.K. conceived the idea for the present study, conducted data analysis, and wrote the paper. J.P.C. designed and funded the biobehavioral assessment program.
